# New CSF biomarkers on the block

**DOI:** 10.15252/emmm.201606801

**Published:** 2016-09-12

**Authors:** Charlotte E Teunissen, Lucilla Parnetti

**Affiliations:** ^1^ Neurochemistry Lab and BiobankDepartment of Clinical Chemistry, Neuroscience AmsterdamVU University Medical Center AmsterdamAmsterdamThe Netherlands; ^2^ Centro Disturbi della MemoriaLaboratorio di Neurochimica Clinica, Clinica NeurologicaUniversità di PerugiaPerugiaItaly

**Keywords:** Biomarkers & Diagnostic Imaging, Neuroscience

## Abstract

A growing number of cerebrospinal fluid biomarkers are now available to capture different aspects of Alzheimer's disease. People are increasingly aware that these biomarkers represent a real‐time reflection of pathological mechanisms that are ongoing in the brain. These novel markers can be added to the panel of existing ones like amyloid beta, total Tau and phosphoTau that are currently used for sensitive diagnosis of Alzheimer's disease, either alone or in combination.

These are exciting times for fluid biomarker research for dementia. The classical cerebrospinal fluid (CSF) Alzheimer biomarkers [amyloid β or Aβ_42_, total Tau (tTau) and hyperphosphorylated Tau (pTau)] have proven useful as diagnostic tools and are now being implemented in clinical routine as guiding the diagnosis decision making process. (Pre‐)analytical variation that preoccupied the field for years is now largely tackled by standardization of procedures, adding Aβ_40_ to the repertoire to control for variation in total amyloid, and automation of analytical methods. From the first publication showing that Aβ_42_ levels were decreased in Alzheimer's patients, it has taken 20 years of research to reach the current status. Since then, numerous papers have shown that the combination of this marker with tTau and pTau has a good discriminatory value even in early predementia stages. These 20 years of research have yielded important information on their biological significance: for example, decreased Aβ_42_ nowadays tells to us that amyloid pathology is ongoing in the brain. Even in preclinical Alzheimer's disease (AD), Aβ_42_ levels change as early as 15–20 years before any clinical symptoms become apparent. Increased tTau has a stronger relation with disease progression, for example, predicts conversion to dementia in patients with mild cognitive impairment (MCI), the prodromal phase of AD (Blennow & Zetterberg, 2015). The three biomarkers are therefore increasingly used as an objective molecular tool to confirm AD pathology in clinical trials targeting the amyloid cascade, more or less interchangeably with amyloid positron emission tomography (PET). Even though partly invasive, the lumbar puncture (LP) necessary to obtain CSF is a safe procedure—the rate of severe postpuncture complaints is as low as 1% (Duits *et al*, 2016) and is generally well accepted in memory clinics. The great value of the CSF biomarkers as unbiased measures that reflect ongoing pathology legitimates the performance of the LP.

But there are new kids on the block! Novel biomarkers are entering the field, especially synaptic proteins, such as neurogranin, and likely more of these will come out soon. CSF neurogranin levels are increased in AD, as first reported in 2010 using a mass spectrometry analysis (Thorsell *et al*, 2010), which was followed by two independent groups end 2015 who further used and developed immune assays (Kester *et al*, 2015; Kvartsberg *et al*, 2015). Now a great interest is devoted to this molecule, as the added value of neurogranin is that it possibly reflects synaptic degeneration, an aspect not mirrored by the classical AD biomarkers. Interestingly, neurogranin is not related to Aβ_42_ but shows a clear correlation with cognitive impairment (Kvartsberg *et al*, 2015). Data available so far indicate that neurogranin is highly specific for AD; in fact, neurogranin levels in other dementia patients are similar (Wellington *et al*, 2016). However, the strong correlation of neurogranin with CSF levels of tTau (*r* = 0.8–0.9) is puzzling, since tTau is an aspecific marker of neuronal damage in neurological diseases (Kester *et al*, 2015).

Another new biomarker is neurofilament light (NfL), a protein likely released from neurons during acute axonal damage. It is not *per se* a very novel biomarker as the first report about NfL CSF levels was published in 2003. However, since then, many studies have been carried out pointing to a few clear advantages in analyzing NfL levels: (i) There is a large body of consistent results in the literature, showing elevated levels in many neurological diseases (Olsson *et al*, 2016). These data could be employed to define reference ranges, for example, (ii) a reliable assay is available, though some adaptations to improve the relatively large inter‐assay variations are needed (which could be solved by developing reference materials and methods); (iii) NfL can be also analyzed in blood, as it has now been shown by several independent technologies (Norgren *et al*, 2005; Gisslen *et al*, 2016). It is important to note that there is a strong correlation between blood and CSF levels for NfL, suggesting that this biomarker could easily be analyzed in the blood compartment. Thus, NfL can be used to monitor treatment effects for several neurological diseases, both in humans and in animal models of amyloid or α‐synuclein proteinopathies (Bacioglu *et al*, 2016) and experimental autoimmune encephalitis (Norgren *et al*, 2005).

What is the added value of all these biomarkers for Alzheimer's dementia? This was the research question addressed by Mattsson *et al* in this issue of *EMBO Molecular Medicine* (Mattsson *et al*, 2016).

Amyloid positivity was in this study treated as a state marker, and the authors hypothesized that patients showing the earliest symptoms, that is, subjective cognitive decline (SCD), first become amyloid positive, and hence, amyloid‐positive SCD patients are included as a distinct group in the study. During disease progression, amyloid‐positive patients develop MCI and ultimately clinical AD. MCI patients that are not amyloid positive are by definition not of the Alzheimer type and thus can be seen as a separate group. The authors studied the levels of tTau, neurogranin, and NfL in these distinct groups. The results showed that (i) optimal discrimination between AD and SCD is obtained when tTau is combined with NfL, while neurogranin does not offer added value here; (ii) the amyloid‐positive patients have higher tTau and neurogranin levels, a pattern that is consistent over disease development, while NfL levels did not depend on amyloid status, as schematically represented in Fig 1; (iii) levels of all three biomarkers were related to hippocampal atrophy and expansion of ventricle volume, but for tTau and neurogranin only specifically in amyloid‐positive patients, whereas the correlations were independent of amyloid status for NfL. Thus, the amyloid status defines the pattern of changes observed in these progression biomarkers. As illustrated by Mattsson and colleagues, CSF is an incredible potential source to gain detailed information on the pathophysiology of diseases of the central nervous system.

**Figure 1:**
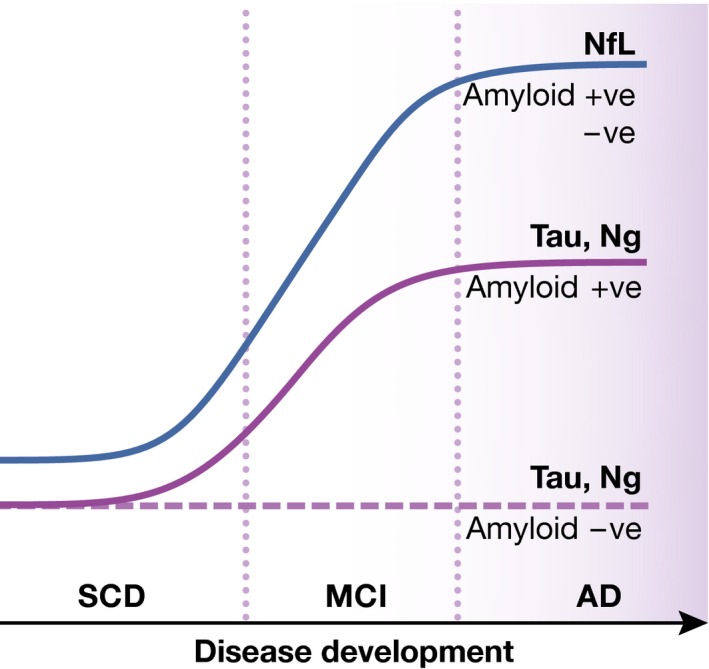
NfL, Ng, and Tau biomarker protein changes during Alzheimer development in relation to amyloid status NfL: neurofilament light; Ng: neurogranin; SCD: subjective cognitive decline; MCI: mild cognitive impairment; AD: Alzheimer's disease.

Because several biomarkers have proven useful, and with the emergence of important consortia such as the Society for Neurochemistry and Clinical CSF analysis (http://www.neurochem.info, see Box 1), clinical routine is well opened to include novel neurological markers. We expect that only a few years will therefore be needed to see this clinically important implementation.

We are living in a new era in which clinical neurology relies on CSF for implementing early diagnosis of neurodegenerative diseases. This is a mandatory step in view of the next availability of disease‐modifying therapies, which, by definition, can bring significant advantages but only if our diagnosis is timely and precise.

**Box 1: The CSF Society**

The Society for Neurochemistry and Clinical CSF analysis (http://www.neurochem.info) is a network of experts, founded in 2015, as a spin‐off of projects such as BIOMARKAPD, SOPHIA, and BioMS‐network, which were all aimed at standardizing and developing guidelines for clinical implementation of CSF biomarkers. This society is a typical body to consult for the development of clinical guidelines incorporating CSF biomarkers. The Society moreover organizes symposia and regional courses to introduce neurologists and neurochemists into CSF as a diagnostic tool, addressing questions such as how to interpret basic CSF analysis, how to perform a lumbar puncture, how to interpret abnormal cellular CSF findings, and the state of the art of CSF biomarker use for several neurological diseases.
